# Impact of Kolb’s experiential learning theory-based nursing on caregiver burden and psychological state of caregivers of dementia patients

**DOI:** 10.1515/med-2025-1157

**Published:** 2025-03-06

**Authors:** Yue Xu, Jingzheng Lu, Xinting Yu, Heguo Ding

**Affiliations:** Department of Psychiatry, Huzhou Third Municipal Hospital, The Affiliated Hospital of Huzhou University, Huzhou, Zhejiang, China; Geriatrics Department, Huzhou Third Municipal Hospital, The Affiliated Hospital of Huzhou University, Huzhou, 313000, Zhejiang, China

**Keywords:** dementia patients, caregivers, Kolb theory, care burden, anxiety, depression

## Abstract

**Background:**

Family caregiving for dementia patients is a challenging task, with profound effects on caregivers’ physical and mental well-being. The purpose of this study was to explore the impact of Kolb’s experiential learning theory on the caregiving burden and psychological status of caregivers for dementia patients.

**Methods:**

This study enrolled 110 caregivers of nursing dementia patients. These caregivers were divided into two groups: a control group (*n* = 55) and an intervention group (*n* = 55). The control group received routine care, while the intervention group received nursing based on Kolb’s experiential learning theory in addition to routine care. Changes in caregiving burden and psychological status were compared between the two groups before and after the intervention.

**Result:**

The results showed that after the intervention, the intervention group’s caregivers had significantly lower scores on the caregiver burden inventory dimensions and total score compared to the control group (*P* < 0.05). Additionally, the intervention group’s self-assessment scores on the self-rating anxiety scale and self-rating depression scale were also lower than those of the control group (*P* < 0.05).

**Conclusion:**

Kolb’s experiential learning theory has a positive impact on alleviating the caregiving burden and reducing anxiety and depression among family caregivers of dementia patients.

## 1 Introduction

“Caregiver burden” refers to the physical, psychological, social, and economic challenges faced by family members while caring for elderly individuals with disabilities [1]. As the health condition of dementia patients gradually deteriorates, their demands and dependence on caregivers increase, resulting in a heavier burden for the caregivers [2]. Over time, caregivers continually confront the worsening condition of their loved ones, which significantly impacts their psychological well-being [3]. In our country, dementia patient care is primarily carried out by family caregivers, but the current state of caregivers’ physical and mental health and caregiving skills is not optimistic [4]. Research indicates that nearly half of caregivers lack essential caregiving knowledge, leading to delays in early diagnosis and intervention for dementia patients [5].

In 1984, the renowned American educator Kolb introduced the Experiential Learning Theory [6], which posits that learning occurs when individuals acquire and transform experiences. According to Kolb, knowledge formation results from “grasping” and “transforming,” enabling better understanding and application of knowledge. While Kolb’s theory has been applied to various patient populations in medical and nursing fields, there is a lack of research on its application to dementia patients’ family caregivers. Therefore, this study aims to explore the impact of a nursing learning plan based on Kolb’s theory on caregiver burden and psychological congruence among family caregivers of dementia patients.

## 2 Methods

### 2.1 Study participants

For this research, we enrolled 110 caregivers diagnosed with dementia in the Department of Psychiatry, Huzhou Third Municipal Hospital, the Affiliated Hospital of Huzhou University, from April 2022 to August 2023. The work was carried out in accordance with the Declaration of Helsinki with the research approved by the Ethics Committee of Huzhou Third Municipal Hospital, the Affiliated Hospital of Huzhou University, under approval No. 2022-064. All participants provide a written informed consent in compliance with the ethics regulations. The participants were divided into control and intervention groups using a random number table method (*n* = 55, respectively). Inclusion criteria for patients were as follows: (1) clinical diagnosis of dementia with some communication ability and (2) age ≥60 years. Exclusion criteria were: (1) patients with critical conditions requiring intensive care unit (ICU) treatment during the intervention period or with fatal outcomes and (2) patients with severe psychiatric symptoms unable to follow instructions.

### 2.2 Inclusion criteria for caregivers

Caregivers must be 18 years of age or older. Caregivers should be primary caregivers (if the patient has multiple caregivers, the order of preference is spouse, parents or children, and others). The duration of caregiving must be at least 3 months, with an expected continuation of care for at least 6 months. Caregivers should have at least an elementary school education level. Participation in the study is voluntary and requires informed consent.

### 2.3 Exclusion criteria for caregivers

Caregivers with congenital or acquired hearing impairments or communication difficulties due to accidents or other reasons should be excluded. Caregivers with psychiatric disorders or cognitive impairments and caregivers currently participating in other research projects should be excluded either. In addition, caregivers whose dementia patients are critically ill and require ICU treatment or have a fatal outcome during the intervention period and who are unable to complete intervention activities due to personal reasons should be excluded.

### 2.4 Intervention study

Both the control group and the intervention group underwent a 4-week intervention.

For the control group, routine nursing care was provided to family caregivers. Disease-related knowledge was imparted primarily through one-on-one education, supplemented by relevant health education manuals. The content covered various aspects of dementia patient care, including basic care, safety measures, medication management, emotional support, and cognitive function care.

**Figure 1:**
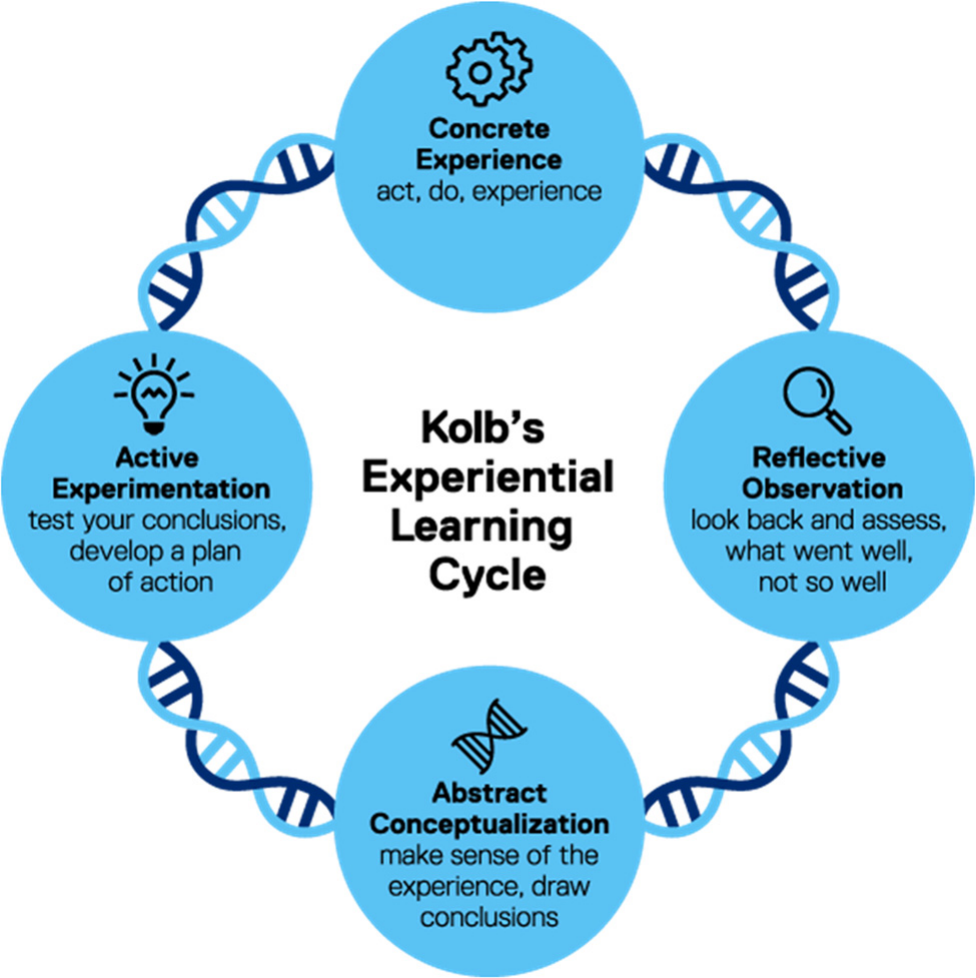
The model graphic of Kolb’s experiential learning.

For the intervention group, a nursing learning plan based on Kolb’s theory was added to the control group’s routine. Specific measures included:

1. Preparation: Before the intervention, data on general patient information, caregiver burden, and psychological status were collected to create personalized learning plans.
2. Personnel training: Standardized training was provided to group members, covering Kolb’s theory, intervention measures, and relevant precautions. Roles within the group were clearly defined.
3. Implementation of nursing plan based on Kolb’s theory: Kolb’s experiential learning theory is a cyclical process that includes four stages (Figure 1): concrete experience, reflective observation, abstract conceptualization, and active experimentation. Learners first start the learning process through concrete experiences, then reflect and observe these experiences, abstract concepts, and theories from them, and finally, apply these concepts to new contexts through active experimentation, thus starting a new cycle.
  In detail, this method is conducted twice a week for 60 min each time. The nursing researcher played the role of a facilitator in the Kolb theory learning plan, introducing the day’s schedule and important notices to family caregivers. Stage 1 (concrete experience): caregivers were asked to play three roles based on case studies: dementia patient, dementia caregiver, and observer. After role-playing, caregivers interacted freely with simulated dementia patients. The nursing researcher created a friendly atmosphere to facilitate easy conversation. Stage 2 (reflective observation): after the role-playing activity, caregivers gathered in a meeting room to reflect on their performance and discuss challenges encountered in daily patient care. They focused on what dementia patients had said to enhance empathy. The nursing researcher encouraged caregivers to express themselves bravely. Stage 3 (abstract conceptualization): Through discussions with the nursing researcher, caregivers gained a broader and deeper understanding of their experiences. They applied the learned nursing knowledge to daily care activities for dementia patients. For instance, if a caregiver reported that a dementia patient repeatedly shared the same story, the nursing researcher provided positive feedback on how to patiently listen to the patient’s repetitive sharing. Additionally, caregivers were advised to introduce new topics to expand conversations and elicit new stories from dementia patients. The nursing researcher acted as an educational guide, providing guidance and suggestions. Stage 4 (active experimentation): Caregivers applied the knowledge acquired in the first three stages to actual dementia patient care scenarios. The nursing researcher helped caregivers face and cope with new challenges.
4. Timely summaries and feedback: Research team members actively participated in and documented the implementation according to the intervention plan. After each intervention, summaries and feedback were provided, and problems were promptly analyzed and resolved.

### 2.5 Observation indicators

Caregiver burden: Assessed using the caregiver burden inventory (CBI) [7]. This inventory comprises 24 items, covering multiple dimensions of burden faced by family members during the care of elderly individuals with disabilities. The dimensions include physical burden, emotional burden, social burden, time dependency burden, and developmental restriction burden. Each item is rated on a 5-point scale (0–4), resulting in a total score ranging from 0 to 96. Higher scores indicate a heavier burden borne during the caregiving process.

Psychological state: Evaluated using the self-rating anxiety scale (SAS) and the self-rating depression scale (SDS). These scales were developed by Zung in the 1970s [8,9], consisting of 20 items and a Likert 4-point rating system. Practically, higher scores indicate more severe anxiety and depression symptoms in patients.

### 2.6 Statistical analysis

We used SPSS 21.0 statistical software for data analysis. Descriptive statistics were employed to present data in the form of mean ± standard deviation, frequencies, and proportions. For the baseline categorical data, we used the chi-square test (*χ*²). For comparison of caregiver burden and psychological states between the two groups, a paired sample *t*-test was applied to investigate the different intervention outcomes. A significance level of *P* < 0.05 was considered statistically significant.

## 3 Results

### 3.1 The baseline characteristics of the studied caregivers

To perform an objective judgment on the impact of Kolb’s Experiential Learning Theory, we should first exclude the influence of participants’ baseline characteristics prior to downstream analysis. As shown in Table 1, there was no statistically significant difference in the general characteristics between the two groups (*P* > 0.05), including factors such as gender, age, disease duration, nursing duration, and education level.

**Table 1:** The baseline characteristics of the study participants

Item		Control group (*n* = 55)	Intervention group (*n* = 55)	*χ* ^2^/*t* Value	*P*-value
**Patients**					
Gender (*n*)	Male	27	28	0.036	0.849
	Female	28	27		
Average age (years)		70.22 ± 6.37	70.11 ± 6.15	0.092	0.927
Disease duration (years)		4.01 ± 1.23	3.88 ± 0.97	0.615	0.540
Disease severity (*n*)	Mid-stage	40	43	0.442	0.506
	Late stage	15	12		
**Caregivers**					
Gender (*n*)	Male	22	25	0.334	0.563
	Female	33	30		
Average age (years)		58.13 ± 7.62	58.21 ± 7.73	0.066	0.948
Education level (*n*)	Junior high school or below	25	28	0.328	0.567
	Technical secondary school or above	30	27		
Relationship with patient (*n*)	Spouse	24	26	1.171	0.557
	Children	21	23		
	Other	10	6		
Caregiving duration (years)		3.42 ± 1.11	3.51 ± 1.05	0.437	0.663

In addition, a comparison of basic burden levels was also made between caregivers and ordinary people (Table 2), which shows that the caregivers were under higher pressure than ordinary people were.

**Table 2:** The comparison of basic burden levels between caregivers and ordinary people

Groups	Cases	SAS	SDS
Caregivers group	*n* = 110	48.99 ± 5.13*	46.78 ± 5.30*
Ordinary people group	*n* = 110	28.66 ± 5.89*	32.19 ± 4.61*

Note: *P < 0.05.

### 3.2 The intervention group shows reduced caregiver burden

Before the intervention, there was no statistically significant difference in the CBI scores between the two groups (*P* > 0.05). After the intervention, the intervention group’s caregivers had lower CBI scores (*P* < 0.05) across various dimensions, including Physical Burden, Emotional Burden, Social Burden, Time Dependency Burden, and Developmental Restriction Burden. In addition, the total score of the intervention group, compared to the control group displays lower CBI scores with statistically significant differences (*P* < 0.05) (Table 3).

**Table 3:** Comparison of caregiver burden between the two groups

Group	Time	Physical burden	Emotional burden	Social burden	Time dependency burden	Developmental restriction burden	Total score
Control group (*n* = 55)	Before intervention	6.36 ± 1.12	7.89 ± 1.43	5.46 ± 0.94	12.85 ± 1.57	11.48 ± 1.53	48.77 ± 5.76
	After intervention	5.85 ± 0.98*	6.73 ± 1.38*	4.77 ± 0.82*	11.73 ± 1.48*	10.35 ± 1.42*	39.73 ± 5.32*
Intervention group (*n* = 55)	Before intervention	6.41 ± 1.10	7.84 ± 1.47	5.39 ± 0.92	12.83 ± 1.52	11.49 ± 1.57	48.34 ± 5.85
	After intervention	5.01 ± 0.86*^,#^	5.65 ± 1.31*^,#^	4.01 ± 0.70*^,#^	10.33 ± 1.36*^,#^	9.23 ± 1.24*^,#^	37.81 ± 3.92*^,#^

Note: In comparison to “Before Intervention Group,” *P < 0.05: In comparison to “Control Group” and ^#^ *P* < 0.05.

### 3.3 The psychological state of the intervention group is ameliorated

To investigate the psychological state of the participants in a quantitative manner, SAS and SDS scores were recorded for the analysis. As is illustrated in Table 4, before the intervention, there was no statistically significant difference in the SAS and SDS scores between the two groups (*P* > 0.05). Whereas after the intervention, SAS and SDS scores of the intervention group were significantly reduced than those of the control group (*P* < 0.05).

**Table 4:** Comparison of psychological states between the two groups

Group	SAS		SDS	
	Before intervention	After intervention	Before intervention	After intervention
Control group	58.81 ± 7.29	48.99 ± 5.13*	56.52 ± 5.89	46.78 ± 5.30^a^
Intervention group	60.10 ± 7.47	42.76 ± 4.82*	58.03 ± 6.00	40.26 ± 4.81^a^
*t*-value	0.897	6.432	1.299	6.498
*P*-value	0.353	<0.001	0.203	<0.001

Note: * indicates statistical significance (*P* < 0.05) within the group before and after intervention.

## 4 Discussion

Alzheimer’s disease, commonly referred to as “senile dementia,” is the most prevalent type of dementia, accounting for approximately two-thirds of all cases. It encompasses various subtypes, including vascular dementia, temporal lobe dementia, and several rare forms of dementia [10]. Globally, around 47 million people suffer from dementia, with age being the primary risk factor for its development. Caring for family members affected by dementia poses a long-term and challenging task [11]. In the middle and later stages of dementia, patients experience cognitive impairments and memory decline, often accompanied by emotional instability, which can lead to conflicts with caregivers. Among this group of family caregivers for dementia patients, a sense of coherence has been shown to predict health-related quality of life, caregiving burden, and caregiving capacity [12].

The nursing learning plan based on Kolb’s theory is an educational approach rooted in experiential learning, which can be traced back to the Experiential Learning Theory proposed by American psychologist David Kolb in the 1970s [13]. According to Kolb, individuals acquire knowledge and skills through a process that involves practical experience, observation, and reflection, which they then apply in real-world contexts [14]. The nursing learning plan, grounded in Kolb’s theory, has been validated in various nursing practice domains and has been shown to positively impact nursing professionals’ competence and care quality [15]. This approach emphasizes active learner participation and experiential engagement [16]. Learners immerse themselves in nursing scenarios through practice and role-playing, leading to a deeper understanding and practical application of nursing knowledge. Additionally, reflection and discussion play a crucial role. By reflecting on observations and abstract concepts, learners enhance their understanding of experiential learning and expand their knowledge through sharing and dialogue. Furthermore, the nursing learning plan based on Kolb’s theory underscores the importance of practice and application. Learners actively apply acquired knowledge to real-world situations, thereby enhancing their nursing skills and confidence [17].

The research results indicate that the intervention group had lower scores in various dimensions of caregiver burden, including physical burden, emotional burden, social burden, time dependency burden, and developmental restriction burden, compared to the control group (*P* < 0.05). This suggests that the nursing learning plan based on Kolb’s theory can assist caregivers in better coping with the various burdens associated with caregiving, which aligns with the findings of the other study [18]. Additionally, caregivers in the intervention group experienced significant relief from negative emotions. Previous research has also demonstrated that Kolb’s theory can provide effective and systematic learning plans and strategies for patients and their caregivers, leading to professional guidance from healthcare providers [19]. This not only expands social resources for caregivers but also helps alleviate anxiety and negative emotions, consistent with the results of this study. Besides, it was observed in the study that there was no significant difference in nursing skills before intervention, but after applying Kolb’s experiential learning theory, the nursing skills of the intervention group were improved. Therefore, implementing a nursing learning plan based on Kolb’s theory enables caregivers to better understand and adapt to the needs of dementia patients, fostering stronger emotional connections and reducing negative emotions, ultimately alleviating stress and burden during the caregiving process.

The effectiveness of nursing learning plans based on Kolb’s theory primarily might stem from two reasons. Firstly, it enhanced the understanding and application of nursing knowledge. Through role-playing, caregivers gain firsthand experience of dementia patients’ feelings and challenges, thereby enhancing empathy toward dementia patients. The reflection and abstract conceptualization stages help caregivers gain a deeper understanding of their experiences and apply the acquired knowledge to practical nursing care. Second, the application of knowledge in real contexts was improved. The active experimentation stage assists caregivers in applying learned knowledge to actual situations, thereby boosting nursing skills and confidence.

However, implementing nursing learning plans based on Kolb’s theory faces certain challenges. On the one hand, the implementation of the learning plan requires sufficient resources and support, including support for training instructors, educational materials, and scheduling. On the other hand, caregivers’ engagement and motivation are also necessitated. Caregivers may encounter limitations in terms of time, energy, and resistance to new learning methods. Encouragement and appropriate support are crucial to foster active participation and motivation among caregivers. As for the weakness of this study, lacking investigation of the caregivers from different cultural backgrounds may lead to a one-sided conclusion. Also, adopting merely Kolb’s theory may not be the optimal strategy. For future research, we recommend further exploring the applicability and effectiveness of nursing learning plans based on Kolb’s theory across different cultural and social contexts. Additionally, combining Kolb’s theory with other educational methods and strategies could benefit clinical outcomes.

In sum, nursing learning plans based on Kolb’s theory have a positive impact on alleviating caregiver burden and improving the psychological well-being of family caregivers of dementia patients. By reducing the burden of care and enhancing understanding of dementia patients, as well as fostering stronger emotional connections, this plan is worthy of clinical implementation and promotion.

## References

[1] Riffin C, Van Ness PH, Wolff JL, Fried T. Multifactorial examination of caregiver burden in a national sample of family and unpaid caregivers. J Am Geriatr Soc. 2018 Nov;67(2):277–83. 10.1111/jgs.15664. PMC6367031 30452088

[2] Lau JH, Abdin E, Jeyagurunathan A, Seow E, Ng LL, Vaingankar JA, et al. The association between caregiver burden, distress, psychiatric morbidity and healthcare utilization among persons with dementia in Singapore. BMC Geriatr. 2021 Jan;21(1):101–10. 10.1186/s12877-021-02014-2. PMC7816438 33468059

[3] Fauth E, Hess K, Piercy K, Norton M, Corcoran C, Rabins P, et al. Caregivers’ relationship closeness with the person with dementia predicts both positive and negative outcomes for caregivers’ physical health and psychological well-being. Aging Ment Health. 2012 Aug;16(6):699–711. 10.1080/13607863.2012.678482. PMC3430821 22548375

[4] Rosgen BK, Krewulak KD, Davidson JE, Ely EW, Stelfox HT, Fiest KM. Associations between caregiver-detected delirium and symptoms of depression and anxiety in family caregivers of critically ill patients: a cross-sectional study. BMC Psychiatry. 2021 Apr;21(1):111–8. 10.1186/s12888-021-03200-7. PMC8035728 33836699

[5] Stansfeld J, Orrell M, Vernooij-Dassen M, Wenborn J. Sense of coherence in family caregivers of people living with dementia: a mixed-methods psychometric evaluation. Health Qual Life Outcomes. 2019 Mar;17(1):211–8. 10.1186/s12955-019-1114-0. PMC6417216 30866961

[6] Murgu SD, Kurman JS, Hasan O. Bronchoscopy education. Clin Chest Med. 2018 Mar;39(1):99–110. 10.1016/j.ccm.2017.11.002. 29433728

[7] Caserta MS, Lund DA, Wright SD. Exploring the caregiver burden inventory (CBI): Further evidence for a multidimensional view of burden. Int J Aging Hum Dev. 1996 Jan;43(1):21–34. 10.2190/2DKF-292P-A53W-W0A8. 8886874

[8] Zung WWK. A rating instrument for anxiety disorders. Psychosomatics. 1971 Nov;12(6):371–9. 10.1016/S0033-3182(71)71479-0. 5172928

[9] Zung WWK. The measurement of affects: depression and anxiety. Mod Trends Pharmacopsychiatry. 1974;7:170–88. 10.1159/000395075. 4153516

[10] Alzheimer’s Association. 2010 Alzheimer’s disease facts and figures. Alzheimer’s Dement. 2010 Mar;6(2):158–94. 10.1016/j.jalz.2010.01.009. 20298981

[11] Moran JA, Rafii MS, Keller SM, Singh BK, Janicki MP. The National task group on intellectual disabilities and dementia practices consensus recommendations for the evaluation and management of dementia in adults with intellectual disabilities. Mayo Clin Proc. 2013 Aug;88(8):831–40. 10.1016/j.mayocp.2013.04.024. 23849993

[12] Andrén S, Elmståhl S. The relationship between caregiver burden, caregivers’ perceived health and their sense of coherence in caring for elders with dementia. J Clin Nurs. 2008 Mar;17(6):790–9. 10.1111/j.1365-2702.2007.02066.x. 18279282

[13] Kolb DA. Management and the learning process. Calif Manag Rev. 1976 Apr;18(3):21–31. 10.2307/41164649.

[14] Konerding U, Bowen T, Forte P, Karampli E, Malmström T, Pavi E, et al. Do caregiver characteristics affect caregiver burden differently in different countries? Am J Alzheimer’s Dis Other Dementiasr. 2018 Dec;34(3):148–52. 10.1177/1533317518822047. PMC10852493 30595033

[15] Hegde A, Chakrabarti S, Grover S. Caregiver distress in schizophrenia and mood disorders: the role of illness-related stressors and caregiver-related factors. Nordic J Psychiatry. 2019 Jan;73(1):64–72. 10.1080/08039488.2018.1561945. 30638102

[16] Healey M, Jenkins A. Kolb’s experiential learning theory and its application in geography in higher education. J Geogr. 2017 Aug;99(5):185–95. 10.1080/00221340008978967.

[17] Lisko SA, O’Dell V. Integration of theory and practice: Experiential learning theory and nursing education. Nurs Educ Perspect. 2010 April;31(2):106–8. 10.1043/1536-5026-31.2.106. 20455368

[18] Yoshimura M, Saiki T, Imafuku R, Fujisaki K, Suzuki Y. Experiential learning of overnight home care by medical trainees for professional development: an exploratory study. Int J Med Educ. 2020 Jul;11:146–54. 10.5116/IJME.5F01.C78F. PMC7870453 32712596

[19] Arakawa H, Anme T. The effect of an experiential learning program on motivations and activity involvement among dementia supporters in Japan. PLOS One. 2020 Dec;15(12):e0244337. 10.1371/journal.pone.0244337. PMC7769555 33370370

